# An evaluation of edge effects in nutritional accessibility and availability measures: a simulation study

**DOI:** 10.1186/1476-072X-9-40

**Published:** 2010-07-27

**Authors:** Emily M Van Meter, Andrew B Lawson, Natalie Colabianchi, Michele Nichols, James Hibbert, Dwayne E Porter, Angela D Liese

**Affiliations:** 1 Division of Biostatistics and Epidemiology, College of Medicine, Medical University of South Carolina, Charleston, SC, USA; 2 Department of Epidemiology and Biostatistics and Center for Research in Nutrition and Health Disparities, Arnold School of Public Health, University of South Carolina, Columbia, SC, USA; 3 Department of Environmental and Health Sciences, Arnold School of Public Health, University of South Carolina, Columbia, SC, USA

## Abstract

**Background:**

This paper addresses the statistical use of accessibility and availability indices and the effect of study boundaries on these measures. The measures are evaluated via an extensive simulation based on cluster models for local outlet density. We define outlet to mean either food retail store (convenience store, supermarket, gas station) or restaurant (limited service or full service restaurants). We designed a simulation whereby a cluster outlet model is assumed in a large study window and an internal subset of that window is constructed. We performed simulations on various criteria including one scenario representing an urban area with 2000 outlets as well as a non-urban area simulated with only 300 outlets. A comparison is made between estimates obtained with the full study area and estimates using only the subset area. This allows the study of the effect of edge censoring on accessibility measures.

**Results:**

The results suggest that considerable bias is found at the edges of study regions in particular for accessibility measures. Edge effects are smaller for availability measures (when not smoothed) and also for short range accessibility

**Conclusions:**

It is recommended that any study utilizing these measures should correct for edge effects. The use of edge correction via guard areas is recommended and the avoidance of large range distance-based accessibility measures is also proposed.

## Introduction

With an increasing interest in the influence of environmental contexts on health behaviors and outcomes, spatial accessibility and availability indices are increasingly applied in epidemiologic studies including those focusing on the built food environment [1-6]. Commonly used availability measures include number or density of outlets, stores, or restaurants in a given location or within a fixed distance of a location. For accessibility, commonly used measures are distance-based; assuming that increased distance acts as a deterrent and reduces the frequency of use of the resource. Frequently, arbitrary administrative boundaries such as Census tracts or block groups are used in lieu of neighborhoods without consideration that resources beyond a given boundary are likely to affect behavior within a spatial unit. Specifically, the effect of edge censoring on such indices has never been fully evaluated. Edge effects occur when the study boundary affects the estimation of a measure and can induce biases which will affect inferences made on the measures [7]. Consider the use of a distance-based measure such as distance to the nearest supermarket. The nearest supermarket may lie outside the study area for locations near the study boundary and thus introducing bias into the spatial measure of distance to the nearest supermarket.

When many observations are close to external boundaries, this effect can be significant. It has been demonstrated that such edge effects can affect the analysis of small area health data [8-10]. This is a form of spatial censoring, where data points outside the study area are not observed. This study evaluates edge effect bias via simulation in applications where accessibility and availability measures are used and recommends approaches to correct or allow for edge effects. This paper is structured as follows. We first outline the measures of interest followed by the simulation design and finally provide results both in terms of contoured maps of error as well as distance profiles of bias.

## Background to Availability and Accessibility Measures

This study evaluated several accessibility and availability measures. Our choice of measures includes those commonly found in the literature of the built food environment [11-18]. Each measure is available at a spatial location within a study area. We define that location as s, which represents the Cartesian coordinates of the location.

### 1. Availability Measures (CI and )

The simplest availability measure we examined is the cumulative index (CI), the count of outlets at a location (or within a pre-defined distance of a location such as a distance buffer, a Census tract, or block group). Hence for a spatial location (s), this is defined as *CI(s) = n(s)*. If we index the location as the i^th ^site then *CI_i _= n_i_*. This measure of availability is frequently used [11-18]. Simple derivatives of this index include density measures, either relative to population [16,19-22] or to area [20,23]. The variance stabilized form of this count is  is often made regularize the variability, and is helpful when there is a need to perform a linear regression model on the square root of the count data [24]. An underlying limitation of the CI is that the spatial unit defines the perimeter of a "neighborhood", i.e. constrains the availability measure to have a "local" nature.

These measures can be computed for a variety of spatial unit sizes. Ultimately the spatial distribution of outlets (or stores or restaurants) is a point process over the study area that may be described by density estimation [25] to provide smoothed local estimates of the density of points. Hence CI is a crude form of a local estimator of density when divided by area. Counts thus are aggregations of outlet locations and maps of counts are smoothed maps of density.

Edge effect censoring can arise with availability measures when counts of outlets are smoothed. For example, averaging of counts within an area will depend on the neighborhood used for the averaging. If part of a neighborhood lies outside the area then some bias will occur in the calculation of the average count near the edge. This is true also for density estimation of point location events [25].

### 2. Accessibility indices (C_p_, distance to the nearest outlet)

Often distance based measures are used to express the idea that potential access to resources diminishes with distance. The distance measured could be road network distance or based on some other relevant distance metric (i.e. Euclidian). The Cumulative Opportunity index (C_p_) is defined in general as  where A is a predefined area within which the distances are measured and s are the location points considered. The distance is measured to all outlets within the area A.

For an indexed location (i):. This measure provides cumulative evidence for accessibility at a spatial location, and can be calculated for special cases such as CP to the nearest outlet, CP for a specified distance buffer, and CP total (calculated over the entire study region). A related measure is the distance to the nearest outlet: *D_i _= d_i _*itself. Both *C_p_(nearest) *and distance to nearest outlet (D_i_) can be extended to include a variety of closeness ('distance to') metrics: nearest, second nearest, third nearest, and the 'sum of distances to' these. For example we could specify a cumulative distance to the 3 nearest outlets, or we could also calculate the cumulative opportunity index for the 2 closest outlets to a location.

Clearly with C_p _measures the smaller the area (A) the more local the measure. One unfortunate feature of the C_p _is that for larger buffers accessibility is being averaged over areas that are distant from the location leading to over smoothing the measure. Hence it is likely to be more informative to use smaller distance buffers in studies of food access.

Edge effect censoring arises with accessibility measures as measures of distance are only available within the study area. This not only potentially skews the distance distribution but also assumes a travel route to food outlets that may not be relevant for any given individual. When a fixed distance buffer is employed and distances are cumulated within the buffer, then the degree of censoring will increase with buffer size. For availability measures these considerations seem less relevant as distance is not usually included in these measures.

## Simulation Study Design

We wish to quantify edge effect bias for these accessibility and availability measures calculated in two spatial environments. We therefore conducted a simulation study to address the nature of the spatial variation of these measures. This study was motivated by and is part of a larger effort on characterizing the built food environment in an eight county region in South Carolina [26].

**Figure 1:**
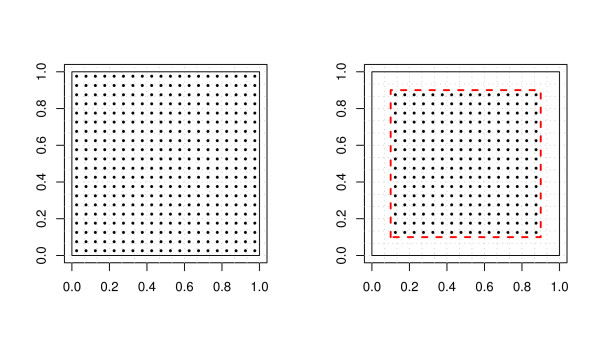
**Simulation Study Design**. Left: Unit square with 15 × 15 grid cells (225 total) and 20 × 20 (400 total) uniformly distributed s location points. Right: Same grid setup with an edge effects boundary represented by the dashed line. Only 256 s location points located inside the edge effects boundary were included.

As is common in evaluation of distance-dependent spatial processes [25] we first defined a unit square study area. This choice allows the evaluation to be carried out without distance scaling and is non-dimensional. The effects of scaling of distance are addressed later. A mesh grid placed over the unit square defines grid cells. Uniformly distributed points placed within these grid cells represent s location points. To assess the effects of edges, we partitioned the study area in two: an internal area and an external guard area. The complement of these areas forms the complete study area (figure 1), where the external guard area is bounded by the dashed and solid black line.

Outlets are then simulated based on model assumptions below. The accessibility measures are then computed for the complete study area. A second set of measures are then computed using only the internal area. Hence for all s location points within the internal area there will be two sets of measures: one computed over the entire study area and the other using only the internal area. Hence the effect of censoring at the edges is captured by this design. Comparison of the two sets of measures allows us to evaluate the degree of bias attributable to edge censoring.

### Model Assumptions

The simulation design is partially based on characteristics of the local food environment and also more general considerations of applicability to a variety of food environment scenarios. To this end we examined outlet densities in an eight county urban and rural area of South Carolina [26]. Large cities are absent, and the average characteristics of outlet density and its variation between rural and urban areas are highlighted. Here we define 'outlet' to mean either food retail store (convenience store, supermarket, gas station) or restaurant (limited service or full service restaurants). Initial simulations considered total stores and restaurants and assumed an outlet density with mean 14.8 and standard deviation of 13.5 per census tract. These summary values correspond to the South Carolina study which identified 2219 food outlets covering 150 census tracts.

We assumed that the study area was divided into a fine tract grid and then we uniformly distributed 400 location points across the unit square grid. Accessibility and availability measures were calculated from the uniformly distributed s location points to outlets in tracts. The outlet densities in our study area [26] suggest overdispersion relative to a Poisson distribution, and initially we examined simulations where outlets were assumed to have a negative binomial distribution in small areas. This however proved to be too simplistic and did not reflect the clustered nature of the outlet distribution. It is often the case that outlets are found in different clustered arrangements in the food environment and so our simulation would be more appropriate if spatial clustering was included in the design.

To accomplish this we designed cluster simulations where a fixed number of cluster centers are assumed and then clustering of outlets around these centers is specified by the parameter *ϕ*. The locations of the cluster centers were randomly simulated using a uniform distribution. To then simulate outlets using this clustering process, we simulated potential outlet locations s* also from a uniform distribution. Then we calculated  where h is a clustering function that has a Gaussian-like form . The term |*s *- *x*_*j*_| is the Euclidean distance between location point s and cluster center *x_j_*. We accepted point s* as a location for an outlet when . *λ(s) *is calculated in the same manner as λ(s*) for all predefined s location points on the grid and *λ*_max _= maximum of *λ(s)*.

Note that these forms are closely related to spatial cluster processes [27]. The cluster centers are fixed in the simulation and outlets are simulated around the centers to mimic aggregation of outlets. While it is clear that in some real cases clusters of outlets occur as linear features related to road systems, it is considerably more difficult to simulate generalizable simulation results from linear features. We believe that clustering modeled around centers can act as an adequate approximation to the real aggregation found, but this assumption has yet to be formally evaluated.

We then used different parameters in the clustering process to distinguish between urban and non-urban areas. We assume there are generally more outlets in urban areas as compared to non-urban areas, and we expect there to be more cluster centers in the urban areas but that the outlets are not as tightly aggregated around each cluster center. The cluster centers could represent a large urban development or shopping area, but we would also expect some locations of outlets to be in the general urban area and not just around the big developments. In contrast, we expect fewer cluster centers in the non-urban areas and that these centers would represent "small" or "large" towns within the non-urban areas. We also expect that the outlets will be more tightly clustered around these cluster centers, and that very few outlets will be in the areas outside the cluster centers. Therefore, we specify a smaller *ϕ *= 0.005 to represent tighter clustering and fewer total outlets in the non-urban areas as compared to *ϕ *= 0.01 and more outlets in the urban simulations.

**Figure 2:**
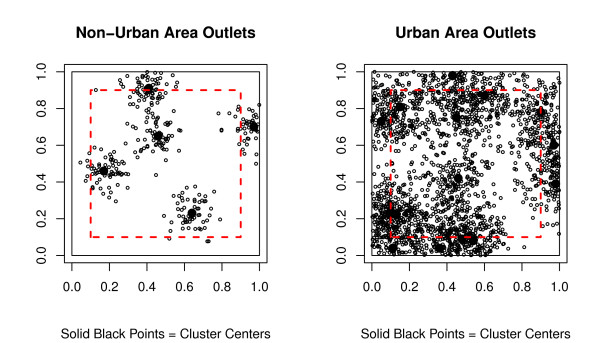
**Examples of simulated outlets using clustering with edge effect boundaries**. The solid black line is the total study area and the dashed line is the internal area, so any outlets outside of the dashed line will not be included in the edge effects analysis. From left to right, the first figure represents a non-urban area with 300 outlets, 5 cluster centers, and clustering parameter *ϕ *= 0.005. The second figure represents an urban area with 2000 outlets, 15 cluster centers, and clustering parameter *ϕ *= 0.01. Solid black dots represent cluster centers and open dots represent outlet locations.

Figure 2 displays examples of both urban and non-urban simulations of outlets using clustering along with the edge effects boundary. This figure illustrates there will be outlets excluded by edge effects, which will create bias in accessibility and availability measures. We expect differences in bias between urban and non-urban areas, as more outlets are excluded in the urban simulation scenario due to the clustering simulation and total number of outlets in the study area.

## Simulation-based results

### Bias and variability

To assess edge effect bias and variability, we calculated the percentage error and absolute bias for each accessibility and availability measure considered. The percentage error and absolute bias for each s location point within the internal boundary area was derived using the calculated spatial measure for the entire grid (internal area + external guard area) versus the calculated spatial measure using only those outlets inside the edge effect boundary (internal area) by the following formula:

and

Tables 1 and 2 display the minimum, median, and maximum values for the median absolute bias among locations that are a specified distance from the edge effects boundary. Table 1 is for an urban simulation with 2000 total outlets and table 2 is for a non-urban simulation with only 300 outlets. Regardless of simulation scenario, as the distance to the edge effects boundary increases, all median absolute bias equal zero except for CP total. Even at small distances, all indices except for CP total have little to no median absolute bias. The absolute bias is much larger in the urban simulation as compared to the non-urban simulation for CP total, and this is intuitive due to the large numbers of outlets located in the external guard area for the urban scenario.

**Table 1:** Minimum, median, and maximum absolute bias for various distances from the boundary in an urban simulation with 2000 outlets

		Median Absolute Bias for Various Spatial Measures						
Distance to Boundary		CI		CP Total	CP Nearest 1	CP Nearest 2	CP Nearest 3	Distance to Nearest Outlet
	Min	0	0	1505.89	0	0	0	0
0.025	Med	6	1.096	2187.42	0	0	0	0
	Max	15	2.161	3158.75	0	2.45	4.61	0
	Min	0	0	1615.79	0	0	0	0
0.075	Med	0	0	1946.53	0	0	0	0
	Max	0	0	2648.79	0	0	0	0
	Min	0	0	1687.62	0	0	0	0
0.125	Med	0	0	1822.69	0	0	0	0
	Max	0	0	2332.78	0	0	0	0
	Min	0	0	1712.66	0	0	0	0
0.175	Med	0	0	1761.12	0	0	0	0
	Max	0	0	2109.84	0	0	0	0
	Min	0	0	1689.10	0	0	0	0
0.225	Med	0	0	1722.31	0	0	0	0
	Max	0	0	1950.82	0	0	0	0
	Min	0	0	1668.79	0	0	0	0
0.275	Med	0	0	1701.53	0	0	0	0
	Max	0	0	1828.89	0	0	0	0
	Min	0	0	1660.19	0	0	0	0
0.325	Med	0	0	1684.29	0	0	0	0
	Max	0	0	1745.45	0	0	0	0
	Min	0	0	1665.01	0	0	0	0
0.375	Med	0	0	1676.79	0	0	0	0
	Max	0	0	1693.05	0	0	0	0

**Table 2:** Minimum, median, and maximum absolute bias for various distances from the boundary in a non-urban simulation with 300 outlets

		Median Absolute Bias for Various Spatial Measures						
Distance to Boundary		CI		CP Total	CP Nearest 1	CP Nearest 2	CP Nearest 3	Distance to Nearest Outlet
	Min	0	0	101.50	0	0	0	0
0.025	Med	0	0	175.69	0	0	0	0
	Max	4	0.91	452.43	0.85	4.47	7.59	0.01
	Min	0	0	110.35	0	0	0	0
0.075	Med	0	0	176.52	0	0	0	0
	Max	0	0	316.41	0	0.17	1.29	0
	Min	0	0	117.62	0	0	0	0
0.125	Med	0	0	170.96	0	0	0	0
	Max	0	0	264.39	0	0	0	0
	Min	0	0	124.30	0	0	0	0
0.175	Med	0	0	166.45	0	0	0	0
	Max	0	0	237.70	0	0	0	0
	Min	0	0	131.19	0	0	0	0
0.225	Med	0	0	164.44	0	0	0	0
	Max	0	0	216.28	0	0	0	0
	Min	0	0	138.00	0	0	0	0
0.275	Med	0	0	162.69	0	0	0	0
	Max	0	0	196.41	0	0	0	0
	Min	0	0	145.58	0	0	0	0
0.325	Med	0	0	161.12	0	0	0	0
	Max	0	0	179.89	0	0	0	0
	Min	0	0	154.39	0	0	0	0
0.375	Med	0	0	160.13	0	0	0	0
	Max	0	0	166.04	0	0	0	0

**Figure 3:**
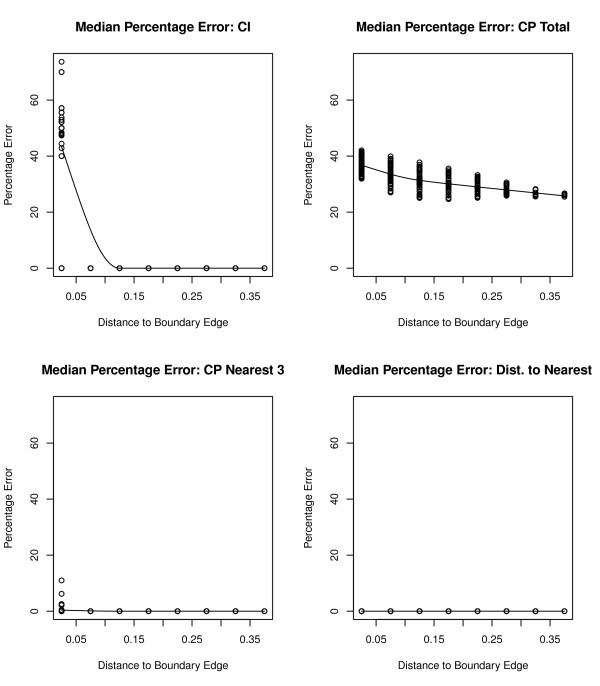
**Plots of the median percentage error for an urban simulation**. Plots of the median percentage error for CI, CP total, CP for the nearest 3 outlets, and distance to the nearest outlet for each s location point over 500 simulations versus the distance from the s location point to edge effect boundary in an urban simulation with 2000 outlets.

Figure 3 displays the median percentage error for various accessibility and availability measures depending on how far the location is from the edge guard boundary for an urban simulation with 2000 outlets. Indices that involve only the first two outlets, such as CP for the 3 nearest outlets and distance to the nearest outlet had median percentage errors equal to zero at even small distances from the edge. CI only saw edge effects at the locations closest to the guard area, but this is expected since the total number of outlets for a location depend on the number of outlets in that particular grid cell. Therefore only grid cells divided by the edge boundary would be affected for this count measure. The poorest performing accessibility statistic in term of median percentage error was CP total. The percentage error is higher at locations closest to the edge boundary; however, we still find median errors of 30% at distances farthest from the boundary. Since CP total is a cumulative measure over the entire study area, the percentage errors are expected and alarming high in this urban simulation.

**Figure 4:**
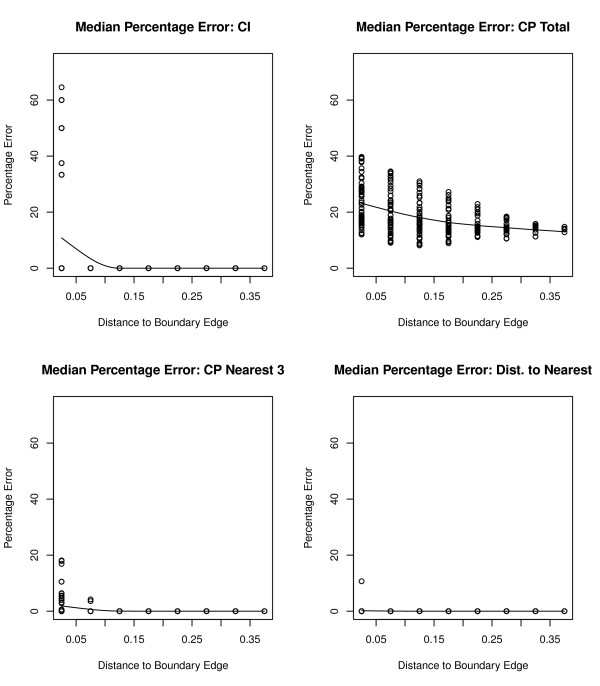
**Plots of the median percentage error for a non-urban simulation**. Plots of the median percentage error for CI and CP total for each s location point over 500 simulations versus the distance from the s location point to edge effect boundary in a non-urban simulation with 300 outlets.

Figure 4 displays median percentage errors for CI, CP total, CP for the nearest 3 outlets, and distance to the nearest outlet for a non-urban simulation with only 300 outlets. Median percentage errors for CI range from 0% to over 60% for locations closest to the edge boundary, but there are fewer overall median percentage errors different from 0% in the non-urban simulation versus the urban scenario. This is attributed to fewer outlets located in the external guard area for the non-urban simulation. Errors for CP to the nearest 3 outlets as well as distance to the nearest outlet are slightly higher in the non-urban scenario. Since there are only 300 total outlets in this simulation, if one of the outlets is located in the external guard area, the next closest outlet may be farther away than one in an urban environment. We find a similar trend regarding the percentage errors for CP total in the rural scenario; however, the errors are generally smaller than what was seen in the urban environment. Since there are fewer outlets in the overall rural simulation as well as in the external guard area, this cumulative CP total is not as affected from edge effects as it is in an urban area.

### Mapped Results and Error Profiles

**Figure 5:**
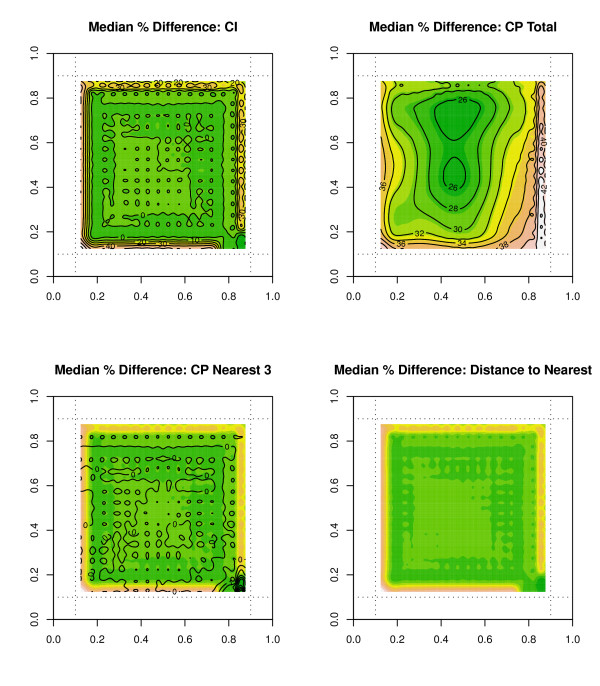
**Contour plots for median percentage errors in an urban simulation**. Contour Plots for Median Percentage Errors at each s location point over 500 simulations for the availability measure CI and accessibility measure CP total for an urban simulation with 2000 outlets.

**Figure 6:**
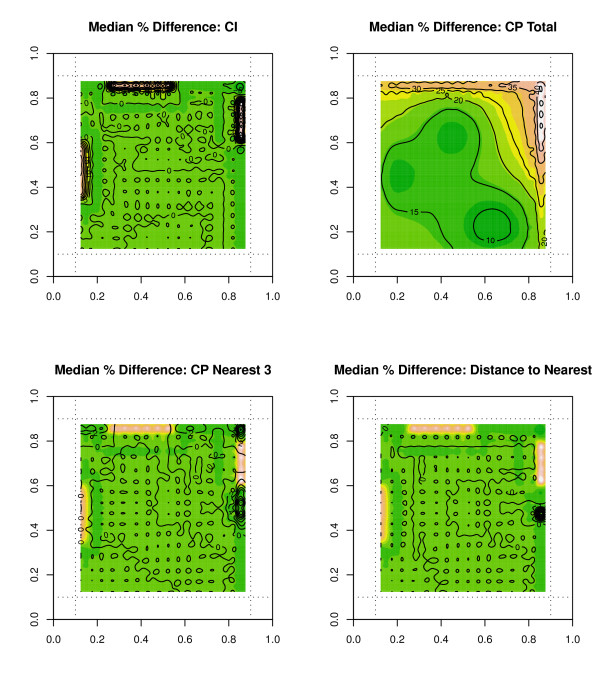
**Contour plots for median percentage errors in a non-urban simulation**. Contour Plots for Median Percentage Errors at each s location pint over 500 simulations for the availability measure CI and accessibility measure CP total for a non-urban simulation with 300 outlets.

We can also present these edge effect percentage errors in contour plots as shown in figure 5 for an urban environment. Once again, we see higher edge effects in areas closer to the edge boundary, and errors for CP total are the highest as compared to other indices. Similarly, figure 6 displays contour plots for the median percentage error over 500 simulations for the non-urban scenario with only 300 outlets. We see increased errors in locations closest to the boundary edge, but this time we see increased errors around the cluster center locations. These cluster centers could represent small or large town environments, and there are few if any outlets located in areas outside of these small town developments.

## Discussion and Conclusions

This paper highlights the importance of edge effects in the analysis of nutritional environment measures. These effects have been of some concern for spatial analysts [7,9,10]. Our simulations demonstrated two sources of bias on analysis results due to edge effects. First, areas close to external boundaries will have additional bias and variance attributable to censoring at the edge. Second, the edge effect can have an overall effect on measure estimation in the map. This means that accessibility measures will be most affected as they use distances as a surrogate for access. Availability measures are less likely to be affected as they are simply local counts of outlets (unless smoothing has taken placed).

The median percentage error showed very small or no edge effect percentage errors for spatial accessibility measures CP to the nearest 1, 2, and 3 outlets as well and the distance to the nearest outlet in both urban and non-urban simulations. However, CP total is greatly affected by edge boundaries regardless of whether the location is close to the boundary edge or not, with over 25% error observed close to the edges and only a marginal decrease to just under 20% at the center of the region. This error is much larger for urban areas than rural areas (see Figure 3 and 4). This suggests that CP total is to be avoided as a measure of choice due to this edge distortion. For availability measures the CI index is greatly affected only at locations next to the edge boundary and is generally robust. If smoothing of CI were performed (e. g, by density estimation or non-parametric regression) then the smoothed estimates will have edge effects.

Remedies for edge effects are available and usually involve some form of weighting system for edge areas. Guard areas either external or internal are useful. External areas would be ideal if that extra information is available as they allow the full estimation of internal measures. Internal guard area is always available in any study but this can limit the usefulness of edge areas as they will be used for estimation of non-edge areas only. Weighting based on proximity to the boundary is also possible, as a compromise between internal guard areas and no compensation. From this study it appears that considerable bias appears in the estimates at or close to boundaries. Clearly the use of guard areas would be recommended in any study. The size of such areas would be important to choose carefully.

The implication of this edge effect is clear. When CP measures are used then it is more robust to use short to medium range measures (1^st ^to 3^rd ^nearest) than to use CP total. In fact CP total is by far the worst measure for edge bias. The CP total measure has large edge effects while the CI and short range CP measures have relatively minor effects. Confining the study to reporting of internal areas is important, and so we would recommend that short range measures be used with a guard area of around 10% of the study window, this being the approximate cut off for the effects for short range measures.

A further set of measures that combine accessibility with availability are gravity measures. These composite measures use distance friction modified by a measure of attraction (such as sales volume, floor space of outlet). Usually they are defined as a ratio of the form *g/d *where g is the measure of attraction of the outlet and d is the distance to the outlet. It is beyond the scope of this study to evaluate these measures. However it is clear that the general behavior of distance-based measures and their behavior at or near boundaries is likely to be found for gravity measures as well in that large distance-based gravity measures will have greater edge biases.

Some limitations and caveats should be mentioned also. First, in our simulation study we only considered a variety of clustered outlet distributions. However, outlets may congregate is more arbitrary clusters or associations (e.g. in linear strip malls or in isolated locations). In addition, the assumption of a Euclidean distance measure may be criticized. This is reasonable in a simulation as we cannot hope to represent the arbitrary network distances of real outlet attraction paths. The statistics we have examined are invariant to these transformations of metrics.

In general our test statistics, and our Monte Carlo limits are robust to scale change and ranges of configurations which at least mimic the marginal properties of real outlet configurations. Thus we believe that the results are generalizable to both different spatial scales and distributions. A limitation that we also admit is that we limited our study to spatial summary measures and didn't pursue the application of geostatistical methods to the fields of measures. The decision to do this was made for two pragmatic reasons: summary measures are commonly used and so are more likely to benefit from edge effect evaluation; geostatistical methods are more difficult to apply and it is more difficult to make comparisons of fields between spatial sites.

## Competing interests

The authors declare that they have no competing interests.

## Authors' contributions

EVM designed the simulation and computational aspects of the work for this paper under supervision by ABL and drafted the manuscript. The general orientation of the work and some detail in the simulations reported in this paper are the responsibility of all authors (EVM, ABL ADL, NC, DP, and JH). The GIS mapping aspects were the responsibility of JH and DP. Manuscript drafts were reviewed and edited by all authors listed.
